# Cetylpyridinium chloride direct spray treatments reduce *Salmonella* on cantaloupe rough surfaces

**DOI:** 10.1111/jfs.12471

**Published:** 2018-03-26

**Authors:** Raúl O. Saucedo‐Alderete, Joseph D. Eifert, Renee R. Boyer, Robert C. Williams, Gregory E. Welbaum

**Affiliations:** ^1^ Department of Food Science and Technology Virginia Tech Blacksburg Virginia; ^2^ Department of Horticulture Virginia Tech Blacksburg Virginia

## Abstract

Cetylpyridinium chloride (CPC) solutions (0, 0.5, or 1.0%) were applied to cantaloupe (“Athena” and “Hale's Best Jumbo” cultivars) rind plugs, either before or after inoculation with a broth culture of *Salmonella* Michigan (10^9^ CFU/mL) and held at 37°C for 1 or 24 hr. Rind plugs were diluted, shaken, and sonicated, and solutions were enumerated. Texture quality and color were evaluated over 14 days storage at 4°C after 0 and 1% CPC spray applications. A 0.5 or 1.0% (vol/vol) application of CPC after *Salmonella* reduced the pathogen levels between 2.34 log CFU/mL and 5.16 log CFU/mL in comparison to the control (*p* < .01). No differences were observed in the firmness and color of 1% CPC treated cantaloupes. *Salmonella* concentrations on cantaloupes, treated with 1.0% CPC, were lower after 1 hr storage as compared to 24 hr. And, *Salmonella* on “Athena” surfaces were more susceptible to CPC spray treatments than on “Hale's Best Jumbo.”

**Practical applications:**

Cetylpyridinium chloride (CPC) is the active ingredient of some antiseptic oral mouth rinses, and has a broad antimicrobial spectrum with a rapid bactericidal effect on gram‐positive pathogens. The spray application of CPC solutions to cantaloupe may reduce the level of *Salmonella* surface contamination during production from irrigation water and manure fertilizers and, during food processing by contaminated equipment and food handlers. Since the surfaces of cantaloupes are highly rough or irregular, bacteria can easily attach to these surfaces and become difficult to remove. Appropriate postharvest washing and sanitizing procedures are needed that can help control *Salmonella* and other pathogens on melons, especially on cantaloupes with nested surfaces. A direct surface spray application of CPC may be an alternative antimicrobial postharvest treatment to reduce pathogen contamination of cantaloupe melons, while providing an alternative to chlorine‐based solutions.

## 1 INTRODUCTION

Over the past 20 years, foodborne illness resulting from contamination of raw melons, particularly cantaloupe, has become an increasing concern to consumers, industry, and regulators (USFDA, 2009). Cases of foodborne illness and illness outbreaks caused by *Salmonella* and other pathogenic bacteria, associated with consumption of fruits and vegetables from both domestic and imported sources, have increased over the last two decades (Bowen, Fry, Richards, & Beuchat, 2006; Centers for Disease Control & Prevention (CDC), 2013; USFDA, 2003a). Specifically, during 1973–2011, 19 illness outbreaks caused by the consumption of cantaloupes were reported, resulting in 1,012 illnesses and 215 hospitalizations (Walsh, Bennett, Mahovic, & Gould, 2014). Most of the outbreaks have been linked to poor or inappropriate cleaning and sanitation at the packing houses. Because cantaloupes are grown at ground level, their outer skins can be contaminated with pathogenic and spoilage bacteria during production from irrigation water and manure fertilizers, and during food processing by contaminated equipment and food handlers (Bowen et al., 2006; Mahmoud, 2012).

Produce packing houses utilize water dunk tanks to clean, sort and disinfect these cantaloupes to eliminate debris, soils and bacteria attached to the products. Chlorine and its derivatives are the most widely used disinfectants to sanitize cantaloupes. Fan, Annous, Keskinen, and Mattheis (2009) found that the application of chlorine and other disinfectants such as acidified calcium sulfate (ACS), acidified sodium chlorite (ASC), and peroxyacetic acid (PAA) had a limited effect on the population of *Salmonella*, achieving no more than a 1.5 log reduction of the pathogen from the surface of whole cantaloupes. There are disadvantages to using chlorine and its derivatives. For example, they are affected by organic matter; they are corrosive at high concentrations; they are not stable in diluted solutions and concentrates, and they cannot be stored for a long time without losing their antimicrobial activity. These drawbacks have led to the search for new disinfection alternatives.

One of these alternatives includes quaternary ammonium compounds (QAC's) which are widely used as disinfectants and antiseptics. QACs are more expensive than chlorine and its derivatives, but they have numerous qualities that make them an attractive alternative for washing fruits and vegetables. QACs are less affected by organic matter; are not corrosive except at high concentrations; they are stable even in diluted solutions and concentrates, and can be stored for a long time without losing their antimicrobial activity (Chaidez, López, & Castro‐del Campo, 2007). According to Frier (1971), QACs are the most useful antiseptics and disinfectants. They are sometimes known as cationic detergents. QACs have been used for a variety of clinical purposes (e.g., preoperative disinfection of unbroken skin, application to mucous membranes, and disinfection of noncritical surfaces). QACs are able to promote their own entry by displacing divalent metal cations in outer cell membranes. In addition to having antimicrobial properties, QACs are also excellent for hard‐surface cleaning and deodorization (McDonnell & Russell, 1999)

Cetylpyridinium chloride (CPC) is a quaternary ammonium molecule that is effective at a concentration of 0.5% for reducing *Salmonella* and cross‐contamination in poultry washes. For example, early research showed reductions of up to 2.5 log for *Salmonella* Typhimurium levels on poultry skin and tissues (Breen, Salari, & Compadre, 1997; Kim & Slavik, 1996). CPC has been approved to treat the surface of raw poultry carcasses prior to immersion in a chiller in the United States (USFDA, 2003b, 2004). Additionally, CPC is commonly used as an active ingredient in mouthwash and toothpaste around the world and it is generally recognized as a safe bactericide.

CPC is bactericidal because of its effects on the cytoplasmic membrane of bacterial cells (Russell, 1998; Russell & Chopra, 1996). According to Fletcher (1996), bacterial adhesion occurs in three steps: reversible absorption, primary adhesion, and colonization. The initial reaction between an antibacterial agent and a bacterial cell involves binding to the cell surface. Changes to outer layers may then occur to allow agents to penetrate the cell to reach their primary site of action and the cytoplasmic membrane or within the cytoplasm. The toxicological effects of CPC on bacteria are caused by the CPC absorbing onto the cell well and the cell membrane (Cutter et al., 2000). The degree of damage to bacterial membrane is time and concentration dependent (Kim & Slavik, 1996). The effect on the primary target site may lead to additional, secondary, changes elsewhere in the organism. Such secondary alteration may also contribute to the bactericidal activity of the CPC (Russell & Chopra, 1996).

*Salmonella* spp. have been implicated in many outbreaks of foodborne illness linked to the consumption of fresh fruits, including cantaloupe melons. And, several serotypes of *Salmonella* have been responsible for multi‐state illness outbreaks and other illness cases associated with consumption of cantaloupe (Richards & Beuchat, 2004; Walsh et al., 2014). Because *Salmonella* can attach to rough surfaces and build biofilm complexes, these organisms can be hard to remove using just chlorine and tap water. A direct spray application of CPC could reduce hard to reach bacteria colonies between the netted surfaces of the cantaloupes. The use of CPC by cantaloupe packers could be an alternative postharvest technique to reduce of possibility of *Salmonella* cross‐contamination at the packaging step.

The objectives of this study are to evaluate the efficiency of any microbial reductions in the level of *Salmonella* by direct spray application of CPC on the surface of two cantaloupe cultivars (Athena and Hale's Best Jumbo [HBJ]). Additionally, this study evaluated the color and texture of cantaloupe during refrigerated storage after a postharvest treatment with a CPC spray solution.

## 2 MATERIALS AND METHODS

### 2.1 Preparing bacterial culture and media

Sodium hydroxide solution (0.1 N NaOH) was prepared using 4 g of NaOH pellets (Certified ACS, Beat UN182, Fisher Chemicals, Fisher scientific) in 1 L of distilled water, then allowed to rest for 1 hr, then 0.5 g of Nalidixic Acid (1‐ethyl‐1,4‐dihydro‐7‐methyl‐1,8‐naphthyridin‐4‐on‐3‐carboxylic acid, 99.5%) powder (Acros Organics, 99.5%, Lot A0272062) was dissolved in the NaOH solution; and mixed on a rotated magnetic plate at slow speed. Nalidixic Acid Solution (Nal stock) was stored in a crystal sterilized container, sealed, wrapped in aluminum foil and stored at 2–4°C for a maximum of 60 days.

Twenty grams of Difco Tryptic Soy Agar (TSA); (Becton–Dickinson and Company) was diluted in 500 mL of distilled water, heated, dissolved, and autoclaved at 121°C × 15 min and cooled. Then, 5 mL of 50 ppm Nal stock was added and stirred for 10 min. Agar (“TSA‐Nal”) was poured into sterile petri dishes which were stored at room temperature to be used the next day.

*Salmonella enterica* serovar Michigan, isolated from a cantaloupe illness outbreak, was obtained from Dr. Larry Beuchat at University of Georgia. A culture was made nalidixic acid resistant by consecutive transfers every 24 hr of isolated colonies from Tryptic Soy Agar with increasing concentrations of nalidixic acid until colonies were resistant at a level of 50 ppm. Colonies were added to Tryptic Soy Broth (TSB) tubes (Becton–Dickinson and Company) and incubated at 35 ± 2°C for 24 hr. After growth, colonies were transferred to a small vial and stored for later use. Bacterial cultures were kept frozen in 80:20 glycerol solutions at −75°C. Prior to each experiment, a culture vial was removed from frozen storage and defrosted slowly by hand. A 0.1 mL aliquot of bacterial culture was added to 9.9 mL of TSB and incubated for 24 hr at 35 ± 2°C. A sample was randomly picked from each group to check for viability in the presence of 50 ppm nalidixic acid. For each sample culture of *S*. Michigan, 100 µL were plated on 40, 50, and 60 ppm TSA‐Nal Plates. Only colonies that grew on 50 ppm TSA‐Nal plates were used in subsequent experiments. *Salmonella* identification was confirmed with a biochemical test kit (API 20 E, identification system for Enterobacteriaceae; bioMérieux, Inc., Durham, NC). Only positive broth cultures were used.

### 2.2 Cantaloupe samples

Cantaloupes were transplanted and direct seeded in consecutive summers at the Virginia Tech College of Agriculture and Life Sciences farm facility (Kentland Farm), Blacksburg, Virginia. First, seeds were planted at the greenhouse facility in 72 cell plug trays to obtain small melons transplants. These were transplanted in early June into black plastic mulch after the last frost. A second planting was done by direct seeding through holes into plastic mulch, to harvest the cantaloupes in sequential stages. Irrigation and fertilization was done using drip irrigation tubes under the plastic mulch. Plants were tended twice per week for weed removal, fruit rotation, and to confirm healthy growth. Insecticides were used only (under the Horticulture Department supervision) as a last resort and weeds were removed by hand. Cantaloupes were harvested when the stem part of the fruits was one‐third or one‐half off (slip stage), indicating that the fruits were ripe.

Undamaged cantaloupes were placed in a cleaned and sanitized plastic reusable box and transported to the Food Science and Technology building at Virginia Tech. Cantaloupes were sorted by size, cultivars, maturity, and cleanness. Over‐ripe, small, and damaged cantaloupes were discarded, only whole good ones that did not show physical or insect damage or broken skins were used. Melons were transferred carefully to a clean water tank and debris was removed by hand and using a soft hair brush. Melons were rinsed using clean tap water and allowed to dry at room temperature (20–25°C) for 30 min. Cleaned and sorted melons were placed in dark plastic boxes and stored at 4°C for a maximum of 7 days in a controlled temperature walk‐in refrigerator.

### 2.3 Rind plug samples

Cantaloupes were transferred to a biological safety cabinet at room temperature (20°C) for 2 hr maximum before being sampled and treated. Cantaloupe rind plugs were collected (2.5 cm diameter, 2.5 cm height, weight ∼10.0 g) using a sanitized sterile cork bored plunger and the flesh adhering to the plug was trimmed off using a sterilized stainless steel single use scalpel. Rind plugs were inserted into a sterile sample container where 9.0 mL of Butterfield's Phosphate Buffer (3M, St. Paul, MN) was carefully added at the bottom of the container to prevent the sample from drying out and to preserve humidity.

SKN samples were chosen that were well netted, thick, coarse, and corky, and stood out in bold relief over some part of the surface, the skin color (ground color) between the netting had changed from green to yellowish‐buff, yellowish‐gray, or pale yellow. SCR samples were chosen that had a layer of cells around the stem that softens, yellowish cast rind, and a smooth symmetrical, shallow base dish‐shaped scar at the point of where the stem was attached. For each trial (3), 18 cantaloupes were used to obtain 40 SKN rind plugs samples and 40 cantaloupes were used to obtain SCR ring plugs.

### 2.4 Cetylpyridinium chloride solution treatments

#### 2.4.1 Preparation of CPC solutions

CPC solutions were formulated as the commercially available Cecure product that consists of CPC, as the active ingredient, and food‐grade propylene glycol in a 1:1.5 ratio. Cecure is a registered trademark of Safe Foods Corporation (North Little Rock, AR). CPC (Sigma‐Aldrich, Lot# 100M0211V, C0732‐100G) was diluted in distilled water to concentrations of 0.5 and 1.0% (wt/vol). Propylene glycol (≥99.5%, Sigma‐Aldrich) was added to each solution in a (1.5:1) ratio. Solutions were stored in clear airtight glass containers at room temperature, away from sunlight, until further use. Distilled water was used as a control (0% CPC). Two treatment applications were performed, where (a) bacteria “Salm” were applied first, followed later by a sprayed solution “CPC” treatment (Salm/CPC), and (b) the spray solution treatment was applied first, followed by the bacteria (CPC/Salm).

#### 2.4.2 *Salmonella*—Chemical spray application (Salm/CPC)

Rind plugs were inoculated with 100 µL of a broth culture of *Salmonella* Michigan (∼10^9^ CFU/mL) using a sterile syringe. This broth culture of *Salmonella* was placed dropwise and spread evenly on the surface of the rind plugs. Then the cantaloupe rind plugs were left to stand for 1 or 24 hr, respectively, in an incubator at 35 ± 2°C. Plugs were sprayed, using a commercial bottle atomizer with self‐adjusted spray nozzle, spraying at an angle of 45° to the surface of the rind plugs samples with 10 mL (3 pump sprays) of a CPC (0, 0.5, or 1.0%) solution and left undisturbed for 15 min in a biosafety cabinet before microbiological analysis. Ten rind samples (3 sample treatments + 1 negative control) were enumerated after 1 hr storage and 10 rind samples (3 samples treatments + 1 negative control) were enumerated after 24 hr storage for each of three replications per trial.

#### 2.4.3 Chemical—*Salmonella* application (CPC/Salm)

Rind plugs were sprayed using a commercial bottle atomizer with a self‐adjusted spray nozzle, spraying at an angle of 45° to the samples, with 10 mL (3 pump sprays) of a CPC (0, 0.5, or 1.0%) solution in a biosafety cabinet. After 15 min, rind plugs were inoculated with 100 µL of a broth culture of *Salmonella* Michigan (∼10^9^ CFU/mL inoculated amount) using a sterile syringe. The broth culture of *Salmonella* was placed dropwise and spread evenly on the surface of the rind plugs. Rind plugs were left to stand for 1 or 24 hr in an incubator at 35 ± 2°C. Ten rind samples were enumerated after 1 hr and 10 rind samples were enumerated after 24 hr for each of three replications per trial.

### 2.5 Microbiological analysis

#### 2.5.1 *Salmonella* recovery (Step 1, simple dilution)

Cantaloupe plugs separately were submerged in 90 mL bottles of Butterfield's Phosphate Buffer. Bottles were shaken for 20 s by hand and decimal dilutions were plated on TSA‐Nal using an automated spiral plater (Autoplate 4000 spiral plater; Spiral Biotech, Norwood, MA).

#### 2.5.2 *Salmonella* recovery (Step 2, dilution and sonication)

The plugs diluted in Step 1 were transferred and placed in a new cup with fresh Butterfield's Phosphate Buffer (99 mL) and sonicated at 75 joules (15 watts for 5 s) in three intervals (1:1:1) using a CPX 130 ultrasonic processor (Cole Palmer Instruments, 130 watts, frequency 20 kHz). The ultrasonic probe had a 6 mm (1/4″) titanium and length of 113 mm (Cole Parmer Instruments, model CV18, series # 2011026727).

#### 2.5.3 Enumeration of samples

Dilutions from the cantaloupe plugs were plated on TSA‐Nal using an automated spiral plater (Autoplate 4000 spiral plater; Spiral Biotech, Norwood, MA). Plates were held at 35 ± 2°C for 24 hr. Colonies were enumerated using a ProtoCOL automated colony counter (Microbiology International, Frederick, MD). All samples were plated in duplicate and the experiment was replicated three times. The recovered cell concentrations for each sample enumerated with and without sonication were summed together prior to additional calculations of mean recovery and statistical significance.

### 2.6 Color analysis

Fifteen whole cantaloupes (“Athena”) were sprayed using a bottle atomizer, with a self‐adjusted spray nozzle, with 40 mL of a 0, 0.5, or 1.0% CPC spray solution and stored at 4°C for 14 days. Color measurements were recorded on Day 1, 2, 5, 7, and 14 of storage for three replicate experiments, using a portable Chromameter (Minolta CR‐300, Japan). For each sample, three readings were interpreted using the Hunter CIE L*a*b* (CIELAB) scale, where L* indicates the level of lightness and darkness, the a* value indicates the degree of redness and greenness, and the b* value indicates yellowness and blueness. A combination of these values were reported as Δ*E* which represents an overall color change. The instrument was standardized using black and white tiles previous to each reading, per the procedure described by the manufacturer of the Chromameter.

### 2.7 Texture analysis

Fifteen whole cantaloupes (“Athena”) were similarly sprayed with 40 mL of a 0% (distilled water) or 1.0% CPC spray solution and stored at 4°C for 14 days. These cantaloupes were not additionally tested for color or microbial recovery. On Day 1, 2, 5, 7, and 14 of storage, the firmness of three cantaloupes was analyzed using a TA‐XT Plus, series 10545, texture analyzer (Texture Technology, New York) with a model TA‐23 plunger (½″ diameter, ¼ R end, 3″ tall). The auto trigger was used with 5 g force and a 2.0 mm/s test distance penetration speed. Readings were collected in triplicate.

### 2.8 Statistical analysis

Three replicate experiments were conducted and two samples (SKN or SCR) of each treatment were analyzed for *Salmonella* Michigan at each sampling time. Data were analyzed by randomized complete block factorial design using general linear model (GLM) procedure of Statistical Analysis Software (Version 9.13, SAS Institute, Cary, NC). Significant differences (*p* ≤ .05) in microbial recovery due to CPC treatment, storage time (1 hr, 24 hr) and order of application (CPC/Salm) or Salm/CPC) were determined using Tukey's multiple range test. Additionally, significant differences (*p* ≤ .05) in color measurements and texture (skin hardness) due to CPC treatments after each storage time were determined using Tukey's multiple range test.

## 3 RESULTS

In this study, 0% (control), 0.5%, and 1.0% CPC direct spray treatment solutions were evaluated for reduction of *Salmonella* Michigan on skin rind plugs (SKN) and stem scar rind plugs (SCR) from “Athena” and “HBJ” cantaloupe cultivars.

### 3.1 Athena's cultivar

**Figure 1:**
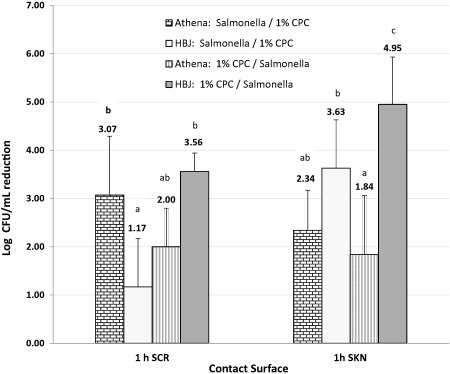
Mean reduction (log CFU/mL) of *Salmonella* from stem scar plugs (SCR) and skin plugs (SKN) from “Athena” (A) and “Hale best Jumbo” (HBJ) after 1 hr incubation periods when CPC spray solution applied before or after the pathogen. Order of application shown in figure legend as: “first”/“second” for each cantaloupe variety. Bar means with the same letter, for each contact surface, are not significantly different (*p* > .05)

Population reductions of *Salmonella* on stem scar plugs (SCR) was approximately 2.0 or 3.1 log CFU/mL when 1% CPC was applied either 1 hr before or after the bacteria, respectively. *Salmonella* was reduced between 1.84 and 2.34 log CFU/mL on SKN plugs when 1% CPC was applied. For both SKN and SCR, *Salmonella* populations were significantly lower (*p* < .05) after 1 hr with each CPC treatment (Table 1, Figure 1). *Salmonella* reduction (from control) after 24 hr storage on SKN ranged from 0.74 to 1.93 log CFU/mL, while the difference in reduction, with no significant differences from control, was less than 0.5 log CFU/mL for SCR plugs.

**Table 1:** Log CFU/mL recovery from stem scar plugs (SCR) and skin (SKN) plugs of “Athena” after 1 hr or 24 hr incubation periods and CPC spray solution applied

	Stem scar plugs (SCR)		Skin plugs (SKN)	
Treatment	1 hr	24 hr	1 hr	24 hr
Order of application				
*Salmonella*, distilled water	7.79 ± 0.78^a^	9.58 ± 0.15^a^	7.51 ± 0.59^a^	9.84 ± 0.16^a^
*Salmonella*, 0.5% CPC	6.20 ± 0.72^ab^	9.40 ± 0.50^a^	6.24 ± 0.09^ab^	8.13 ± 0.49^a^
0.5% CPC, *Salmonella*	4.89 ± 1.10^b^	9.57 ± 0.12^a^	7.25 ± 0.38^a^	9.10 ± 0.83^a^
*Salmonella*, 1% CPC	4.72 ± 1.22^b^	9.15 ± 0.93^a^	5.17 ± 0.83^b^	7.91 ± 0.38^a^
1% CPC, *Salmonella*	5.79 ± 0.80^ab^	9.15 ± 0.24^a^	5.67 ± 1.22^ab^	8.29 ± 1.59^a^

Column means with the same letter are not significantly different.

### 3.2 Hale's Best Jumbo cultivar

Population reductions of the *Salmonella* on SKN rind plugs (4.95 log CFU/mL greater than control) was significantly greater (*p* < .05) when 1.0% CPC was applied 1 hr before the bacteria (CPC/Salm). Additionally, when 1.0% CPC solution was applied after *Salmonella*, the reduction of *Salmonella* on SKN was 3.63 log CFU/mL greater than control. Population reductions of the *Salmonella* on SCR plugs (3.56 log CFU/mL) was also highest when 1% CPC was applied 1 hr before the bacteria (CPC/Salm). *Salmonella* reduction (from control) after 24 hr storage of SKN and SCR rind plugs was <1.2 log cfu/mL, with no significant differences from control, for all combinations of CPC concentration and order of application (Table 2, Figure 1).

**Table 2:** Log CFU/mL recovery from stem scar plugs (SCR) and skin (SKN) plugs of “HBJ” after 1 hr or 24 hr incubation periods and CPC spray solution applied

	Stem scar plugs (SCR)		Skin plugs (SKN)	
Treatment	1 hr	24 hr	1 hr	24 hr
Order of application				
*Salmonella*, distilled water	7.17 ± 0.88^a^	8.72 ± 0.78^a^	7.43 ± 0.48^a^	9.17 ± 0.53^a^
*Salmonella*, 0.5% CPC	5.53 ± 1.24^ab^	7.52 ± 1.63^a^	3.72 ± 0.08^bc^	8.76 ± 0.77^a^
0.5% CPC, *Salmonella*	4.70 ± 0.37^b^	8.32 ± 0.40^a^	4.38 ± 0.43^b^	8.50 ± 0.22^a^
*Salmonella*,1% CPC	6.00 ± 0.32^ab^	8.72 ± 0.19^a^	3.80 ± 0.69^bc^	8.06 ± 0.62^a^
1% CPC, *Salmonella*	3.61 ± 0.38^b^	7.94 ± 0.32^a^	2.48 ± 0.98^c^	8.68 ± 0.90^a^

Column means with the same letter are not significantly different.

Higher log reductions for the CPC/Salm (1% CPC spray first and *Salmonella* inoculation second) treatments demonstrate the significant antimicrobial effects of CPC on bacteria cells in a short period (1 hr). The antimicrobial effects of CPC are dependent on binding to bacterial cells (Caputo, Treick, Griffin, & Farrell, 1975) and bactericidal activity in the presence of serum proteins and at different pH and temperature (Quisno & Foter, 1946). On the other hand, after 24 hr storage, bacterial cells had time to adapt to the environment and population growth increased.

### 3.3 Athena and HBJ cultivars

The log reduction (cfu/mL) of *Salmonella* with 1% CPC spray solution after 1 hr (Salm/CPC) on stem scar rind (SCR) of “Athena” was significantly greater (*p* < .05) than with the 1 hr (Salm/CPC) treatment of SCR on “HBJ” melons. Conversely, the log reduction (CFU/mL) with 1% CPC solution after 1 hr (CPC/Salm) on SCRSCR rind of “HBJ” was higher, but not statistically significant, than (CPC/Salm) on SCR rind of Athena's. For both cultivars, storage of cantaloupes treated with 1.0% CPC solution for 1 hr had a greater effect on reducing *Salmonella* compared to 24 hr treatment.

### 3.4 Texture and color

**Figure 2:**
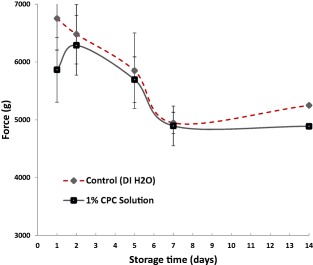
Skin hardness test (force (g) applied) on whole cantaloupes (“Athena”) after 0% (control) or 1.0% CPC spray solution applications and 1, 2, 5, 7, and 14 days storage at 4°C

No significant differences were observed in the hardness of 1.0% CPC treated cantaloupes at 7 and 14 days compared to control. At Day 14, a similar level of force was required to penetrate the skin of the control and CPC sprayed melons (Figure 2).

Color measurements of treated cantaloupes during 14 days storage are summarized in Table 3. While the experiment control melons samples appeared slightly darker than those sprayed with the 1% CPC solution on Day 1 of storage, no statistically significant differences in color parameters were found between 1.0% CPC solution treated cantaloupes and controls throughout all storage days. In addition, the sensory (color and texture) quality of cantaloupes at the end of refrigerated storage did not suffer any major change based on the visual appearance of the outside of intact cantaloupes and their degree of deterioration.

**Table 3:** Mean color measurements after spray application of 0, 0.5, and 1.0% CPC on Athena cantaloupes stored for 14 days at 4°C

	Day 1	Day 2	Day 5	Day 7	Day 14
Distilled water (Control)					
*L* mean	46.99 ± 0.67	45.94 ± 1.17	44.72 ± 1.70	43.11 ± 2.49	43.11 ± 4.24
*a* mean	49.34 ± 0.98	46.63 ± 2.09	48.08 ± 0.67	46.75 ± 1.54	47.50 ± 1.00
*b* mean	52.00 ± 0.69	50.65 ± 0.70	50.29 ± 0.90	48.53 ± 2.80	49.02 ± 2.98
Δ*E*		3.21	2.71	4.46	3.98
0.5% CPC					
*L* mean	43.13 ± 2.62	44.23 ± 2.11	43.26 ± 3.86	42.40 ± 0.89	41.49 ± 1.52
*a* mean	46.75 ± 0.86	46.73 ± 3.56	46.09 ± 1.07	48.55 ± 1.57	45.86 ± 1.18
*b* mean	49.59 ± 1.03	51.55 ± 1.40	48.52 ± 2.85	49.14 ± 1.01	47.54 ± 1.14
Δ*E*		2.25	3.10	2.64	3.53
1.0% CPC					
*L* mean	45.16 ± 0.52	42.20 ± 1.25	43.65 ± 1.30	45.07 ± 0.73	43.19 ± 0.85
*a* mean	44.58 ± 1.68	47.33 ± 1.42	47.31 ± 0.95	47.79 ± 1.24	47.29 ± 1.46
*b* mean	49.55 ± 0.83	48.35 ± 1.21	49.64 ± 0.36	51.94 ± 1.08	49.43 ± 1.67
Δ*E*		4.21	1.99	2.35	3.23

*n* = 3.

*L* = 0 yields black and *L* = 100 indicates diffuse white; spectacular white.

*a* = negative values indicate green while positive values indicate magenta.

*b* =negative values indicate blue and positive values indicate yellow.

Δ*E* = Total color difference.

## 4 DISCUSSION

In this study, we demonstrate that a spray application of CPC solution can reduce *Salmonella* Michigan between 2.3 to 4.9 log CFU/mL. Other researchers also reported at least a 2‐log reduction in microbial populations when applying CPC solutions to produce. Hong, Yanbin, and Slavik (2001) reported that vegetables treated with 0.1 and 0.5% CPC reduced *Salmonella* Typhimurium by 2.37 and 3.15 log CFU/g. Araya‐Rodríguez et al. (2008) found that the use of a 5 s dip in 0.5% CPC significantly improved the microbial shelf life of cantaloupes and Spanish melons when applied either directly to field harvested melons or after the current commercial processing and washing procedures allowing for a 99% reduction in aerobic plate count. And, Beaulieu, Dumas, and Janes (2006) reported that *Salmonella* Montevideo was reduced by 3 log CFU/g on inoculated cantaloupe cubes that were treated with 0.8 or 1.0% CPC and stored for 24 hr.

The effect of CPC treatments on the reduction of bacterial attachment to the rough surfaces of cantaloupes can vary, depending on the contact time and type of netted surface. Application of antimicrobials to produce surfaces may be useful if they can reduce bacterial populations in wash water and reduce cross‐contamination. Lopez, Phalen, Vahl, Robert, and Getty (2016) noted that the use of disinfectants to wash cantaloupe may be most useful to maintain process/wash water free of microbial contaminants and reduce the risk of cross‐contamination when new produce is introduced to the washing sink or tank. In their study, a commercial produce wash was able to reduce *Salmonella* by no more than 1.26 log CFU/cm^2^ on cantaloupe surfaces.

CPC and other sanitizers can be an important alternative to disinfecting produce surfaces, especially where chlorine solutions are ineffective or not permitted. Tan et al. (2015) compared several sanitizers for inactivating *Salmonella* on whole turnips. A 3 min dip into a 200 ppm chlorine solution was similarly effective to a 3 min dip into a 1% (wt/vol) CPC solution for reducing *Salmonella* by 1.55 and 1.43 log CFU/turnip, respectively.

For some test combinations, the log reduction of *Salmonella* was significantly higher when 1.0% CPC, rather than 0.5% CPC, was applied. In some tests, the log reduction with 0.5% CPC was slightly significantly higher when compared to 1.0% CPC. This reinforces the argument that the effects of biocides on bacterial (and, other types of microbial) cells should be examined over a wide range of concentrations (Russell & McDonnell, 2000). Other studies with tomato and cantaloupes inoculated with human pathogens revealed that when the time interval between inoculation and washing with sanitizer agent increased from 1 hr to several days, the efficacy of sanitizer treatment in reducing pathogen populations decreased (Sapers & Jones, 2006; Ukuku & Sapers, 2001). Therefore, the timing of antibacterial chemical applications, along with the chemical concentration, can greatly affect the level of pathogen reduction.

In the United States, CPC has been used in many oral hygiene products long before it was permitted for use on a food (raw poultry processing). Other antimicrobial chemicals, primarily used in mouthwashes or other oral hygiene products, should be considered for food applications for inactivating microbial pathogens. For example, octenidine dihydrochloride is a cationic, bispyridinamine used in oral rinses as an antimicrobial and antiplaque agent in Europe that exhibits antimicrobial activity against a wide range of pathogenic microorganisms. Upadhyay et al. (2016) reported that washing cantaloupe rind plugs in 0.1% octenidine dihydrochloride for 1 min could reduce *Salmonella* by >3 log CFU/cm^2^. Additionally, Saucedo‐Alderete (2013) reported that a spray application of 1.0% delmopinol hydrochloride reduced *Salmonella* concentrations by ∼3.1 log CFU/mL (more than a distilled water control) on cantaloupe skin rind plugs and stem scar rind plugs.

In this study, a CPC spray solution was highly effective for microbial reduction when it was applied after *Salmonella* application for both “Athena” and “HBJ” cultivar melons. Further studies are needed to investigate more fully the mechanisms of inhibition and inactivation of gram‐negative bacteria, such as *Salmonella*, by CPC, and its efficacy on a wider variety of raw foods and in food processes.
